# Unimanual and bimanual motor performance in children with developmental coordination disorder (DCD) provide evidence for underlying motor control deficits

**DOI:** 10.1038/s41598-021-85391-3

**Published:** 2021-03-16

**Authors:** Melody N. Grohs, Rachel L. Hawe, Sean P. Dukelow, Deborah Dewey

**Affiliations:** 1 Department of Neurosciences, University of Calgary, Calgary, Canada; 2 Owerko Centre at the Alberta Children’s Hospital Research Institute (ACHRI), #397, Child Development Center, 2500 University Dr. NW, Calgary, AB T2N 1N4 Canada; 3 Department of Clinical Neurosciences, University of Calgary, Calgary, Canada; 4 Hotchkiss Brain Institute (HBI), University of Calgary, Calgary, Canada; 5 Department of Pediatrics, University of Calgary, Calgary, Canada; 6 Department of Community Health Sciences, University of Calgary, Calgary, Canada

**Keywords:** Neurodevelopmental disorders, Paediatric research

## Abstract

Much of our understanding of motor control deficits in children with developmental coordination disorder (DCD) comes from upper limb assessments focusing on the dominant limb. Here, using two robotic behavioural tasks, we investigated motor control in both the dominant and non-dominant limbs of children with DCD. Twenty-six children with diagnosed DCD (20 males; mean age 10.6 years ± 1.3 years) and 155 controls were included in this cross-sectional study. Participants completed a visually guided reaching task with their dominant and non-dominant limbs and a bimanual object hitting task. Motor performance was quantified across nine parameters. We determined the number of children with DCD who fell outside of the typical performance range of the controls on these parameters and compared the DCD and control groups using ANCOVAs, accounting for age. Children with DCD demonstrated impairments in six out of nine parameters; deficits were more commonly noted in the non-dominant limb. Interestingly, when looking at individual performance, several children with DCD performed in the range of controls. These findings indicate that children with DCD display deficits in motor control in both the dominant and non-dominant limb and highlight the importance of including detailed assessments of both limbs when investigating children with DCD. They also demonstrate the variability in motor control performance evidenced by children with DCD.

## Introduction

Developmental coordination disorder (DCD) is a neurodevelopmental disorder associated with impairments in the acquisition and execution of coordinated motor abilities^1,2^. The marked motor difficulties present in affected individuals greatly interfere with activities of daily living, and have been found to have negative repercussions in academic, cognitive and social domains^3^. Movements are often described as being slow, inaccurate and/or awkward, which in turn lead to functional difficulties in tasks such as tying shoe laces, hand-writing, catching a ball or riding a bike^4^. DCD continues to remain largely under-recognized and under-diagnosed within the healthcare and educational systems^2^; This may in part be related to significant heterogeneity in the symptomology associated with DCD^5^.

Despite its name, the difficulties seen in individuals with DCD extend beyond coordination with widespread sensory and motor deficits being reported throughout the research literature^6,7^. It has been suggested that impairments in motor control may underlie the deficits seen in children with DCD^6^. Motor control is the ability to initiate and produce purposeful, coordinated and precise movements^8^. Various research groups have used reaching and pointing paradigms to investigate different aspects of motor control in children with DCD. Most commonly, participants have been instructed to reach or point with their dominant limb to a switch or a target on a digitizing screen, while measures related to movement time^9–15^ (i.e., reaction time, total movement time, deceleration time) and movement errors^10,11,15,16^ (i.e., number of targets or touchdown errors where the final limb placement is outside the desired target location) were recorded. Other researchers have used motion capture software to investigate reaching with the dominant limb, as it permits the collection of spatial and temporal metrics such as acceleration, velocity and path trajectory^17–21^. Converging results suggest children with DCD have slower reaction times^12,13,17,19^, longer movement and/or deceleration times^9–12,14,15,17,20,21^ and display more errors when making movements^10,11,15–17,19,20^. Additionally, longer and more curved path trajectories^18,20,21^ as well as decreased accuracy and more variability in movement speeds^17,18,20^ have been reported in children with DCD.

Interestingly, very few reaching or pointing studies in children with DCD include assessments of the dominant and non-dominant limbs or bimanual abilities^10,15,22,23^. The few existing studies that have investigated the non-dominant limb^10,15,23,24^ and bimanual abilities^10,22^ have focused on movement time and target errors, and do not include detailed kinematic measures. Results from these studies, and a study investigating bimanual coordination in children with DCD^25^, suggest that deficits may be more prominent in the non-dominant limb and that children with DCD may also show difficulty with inter-limb coupling. Given these findings, studies which include assessments of both limbs (individually and together) as well as detailed kinematic measures, may provide a more comprehensive understanding of the underlying motor control deficits present in children with DCD.

Here we use two robotic behavioural tasks—a visually guided reaching task and a bimanual object hitting task—to investigate dominant and non-dominant limb performance in children with DCD. Robotic technology provides an avenue for objective, reliable and accurate assessment of sensorimotor performance^26,27^; in particular, the visually guided reaching and bimanual object hitting tasks used in the current study have been shown to be clinically valid assessments of motor impairment in adult and pediatric populations with motor difficulties, including stroke^28–30^, cerebral palsy^31^, traumatic brain injury^32^ and epilepsy^33^. Using robotics, we can examine decision making abilities, motor control and planning abilities as well as dominant, non-dominant and bimanual limb performance, all within a fairly rapid assessment time (10 min). Additionally, the bimanual object hitting task used in the current study is novel to a DCD population and allows for the investigation of motor control abilities during a more demanding task (i.e., using both hands simultaneously to intercept moving targets, compared to a traditional reaching task where participants reach to a static target).

Finally, previous research has investigated group differences in motor performance; however, group differences may not reflect the heterogeneity of motor performance seen in children with DCD. Therefore, the current study used data from 155 typically developing control participants^34^ to establish typical performance ranges for each robotic behavioural task, to which the performance of children with DCD were compared at both the individual and group level. We hypothesized that dominant and non-dominant limb performance of children with DCD would fall outside the typical performance range on both the unimanual and bimanual tasks.

## Methods

### Participants

Children with DCD (n = 26) were recruited through developmental and community pediatricians, psychologists and physical/occupational therapists within Calgary, Alberta, as well as through social media. Inclusion criteria were: (1) 8–12 years of age, (2) current diagnoses of DCD by a registered health care provider, (3) the presence of a motor deficit as confirmed by a Total Test score on the Movement Assessment Battery for Children-Second Edition (MABC-II) below the 16th percentile ^35^, (4) typical cognitive performance based on a Full-Scale IQ score $\ge$ 80 on the Wechsler’s Abbreviated Scale of Intelligence-Second Edition (WASI-II), and (6) right-handed (defined as the hand used for writing). Exclusion criteria were (1) pre-term birth (< 36 weeks’ gestation) and (2) the presence of any visual impairment or a severe neuropsychiatric, neurological and/or chronic disorder (i.e., autism spectrum disorder, epilepsy, musculoskeletal disorder) as confirmed by a parent questionnaire, which was used to obtain a detailed medical history of the child. Children with corrected vision (i.e., glasses) were included in the current study as well as children with DCD who had been previously diagnosed with attention deficit/hyperactivity disorder (ADHD), learning disorder (LD), or generalized anxiety disorder (GAD) by a registered health care provider, due to the high co-occurrence with DCD^36^. Parent reports of children with DCD confirmed that 14 children had a diagnosis of ADHD, 10 had a diagnosis of a LD, and 4 had a diagnosis of GAD.

One hundred and fifty-five typically developing (TD) children from a previously recruited cohort^34^ participated in the same robotic assessments and were included in the current study. In short, inclusion criteria for this group were: (1) 6–19 years of age, (2) full term birth (> 36 weeks’ gestation), and (3) the absence of any visual impairment, neurological disorders or sensorimotor deficits as confirmed through a detailed clinical assessment by a registered physiotherapist. All 155 controls were included in our investigation of individual level performance; a subsample of the controls falling between the ages of 8–12 years (n = 57) were included in the final group level analyses that compared performance of children with DCD (n = 26) to typically developing children. This study was approved by the University of Calgary’s Conjoint Health Research Ethics Board (REB18-0183; REB15-0136) and performed in accordance with relevant regulations and guidelines. Participants’ legal guardians provided written informed consent and children provided assent at the time of enrollment.

### Clinical assessment of motor and cognitive function

For children with DCD, motor functioning was assessed by an investigator (MNG), trained and supervised by a registered psychologist (DD), using the Movement Assessment Battery for Children-Second Edition (MABC-II)^35^. The MABC-II is a valid and reliable standardized motor assessment, used to evaluate motor functioning in children and adolescents between the ages of 3–16 years on tasks relevant to daily living (i.e., manual manipulation of small objects, handwriting, catching)^37,38,^ and is commonly used within the DCD literature to assess motor functioning ^39^. It provides standardized scores based on normative data of 1172 children from the United Kingdom (mean population standard score 10 ± 3) and takes approximately 30 minutes to administer. The MABC-II is comprised of three subscales: (1) Manual Dexterity, where the number of errors are counted on a drawing task and participants are timed on a peg placing task as well as a threading lace or building task; (2) Aiming and Catching, where participants were scored out of ten on the number of successful ball catches as well as tosses at a target; (3) Balance, where participants are timed during performance of a one or two-legged balance task, as well as scored on the number of successful heel-to-toe-steps made, up to a maximum of fifteen, and the number of one-legged jumps made across five consecutive mats. Raw scores are converted into age standardized item scores for each subscale item. These standard scores are summed to get an overall component score for each subscale, which is converted into a standard score and a percentile. A Total Test score is calculated by summing each of the subscale component scores and correspondingly calculating a standard score and percentile. A Total Test score below the 16th percentile indicates the presence of motor deficits ^35^.

DCD participants also completed the Wechsler Abbreviated Scale of Intelligence-Second Edition (WASI-II)^40^, administered by the same trained investigator (MNG). The WASI-II is a standardized assessment that provides a valid and reliable measure of intelligence^41^. All four WASI-II subtests were completed (i.e., Block Design, Vocabulary, Matrix Reasoning and Similarities). Full scale IQ scores of < 80 are indicative of cognitive deficits^40^.

### Robotic assessment

**Figure 1:**
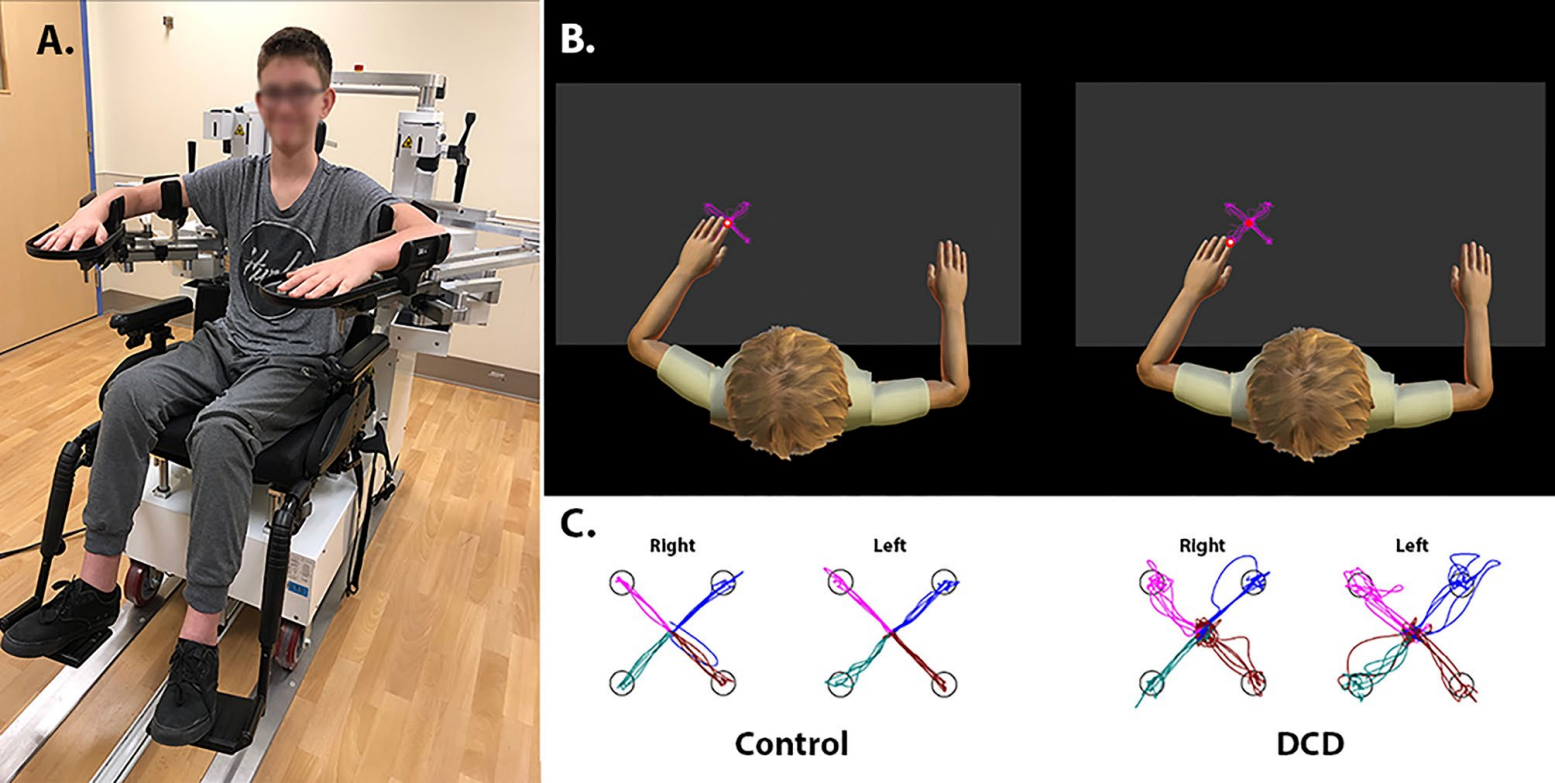
(**A**) Participant shown in the Kinarm exoskeleton robot, equipped with a wheelchair base and arm troughs supporting each arm in the horizontal plane; (**B**) schematic of the Kinarm workspace for the visually guided reaching task. The graphic demonstrates a reaching movement of a DCD participant. The participant’s fingertip begins within the centered red illuminated target. A peripheral target will appear, in which participants will then reach to that target as quickly and accurately as possible. (**C**) Right- and left-hand path trajectories, for the four peripheral spatial targets, are shown for a typically developing control and a DCD participant of similar ages; note that participants arms were visually occluded and hand position feedback was provided by the virtually displayed white dot placed over the tip of the index finger.

Motor performance was assessed using the Kinarm exoskeleton robot (Kinarm, Kingston, Ontario, Canada) at the Alberta Children’s Hospital, Calgary, Alberta, Canada. Participants were seated into a wheelchair base with each arm supported in the horizontal plane by the exoskeleton structure (Fig. 1a; written consent for the publication of an identifying image in an open-access journal was obtained from the participants legal guardian). Adjustments were made for participant height and length of arm reach. Once fitted, participants were wheeled into an augmented reality workstation and a standard calibration procedure was undertaken before beginning the motor tasks.

#### Visually guided reaching task

Participants were instructed to “reach as quickly and accurately as possible” from a fixed central position to one of four peripherally located spatial targets. Targets were 6 cm apart, appearing as red dots on the display screen. Participants arms were visually occluded; however, hand position feedback was provided by a virtually displayed white dot placed over the tip of the index finger (Fig. 1b,c). Participants completed a total of five blocks, each consisting of four randomly generated peripheral target locations. Participants completed the task with their dominant limb first, followed by their non-dominant limb. Task performance was measured at a sampling frequency of 1000 Hz using four selected parameters, which are outlined below. This visually guided reaching task has been described in detail previously^28,34^.

#### Measured parameters

1. *Reaction time (in seconds)*—a measure of the time between the illumination of a peripheral target and the participant’s onset of movement in response to that target.
2. *Initial movement direction error (in degrees)*—the angular deviation between the “desired” movement (straight line from the hand location at movement onset to the peripheral target) and the “true” movement (hand position at movement onset to the hand position after the initial movement).
3. *Difference between speed minima and maxima (in meters/second)*—the calculated difference between local maximum and minimum hand speeds throughout the movement.
4. *Hand path length ratio*—the total distance traveled by the hand between movement onset and offset compared to the actual distance between the targets.

#### Movement onset

To determine movement onset, lower and upper speed thresholds were calculated for each participant; (1) a lower speed threshold was calculated with median hand speed across all trials for 500 ms before end point target illumination, (2) an upper speed threshold was calculated with the 95th percentile of hand speed, again for the 500 ms before end point target illumination. Once the hand had left the start target, the algorithm moved time backwards to the calculated local minimum hand speed, or when the hand speed dropped below the calculated lower speed threshold, this was deemed movement onset for a given reach. For further details on the movement onset algorithm see Coderre et al.^28^ and https://kinarm.com/about-us/ (Kinarm, Kingston, Ontario, Canada).

#### Object hit task

**Figure 2:**
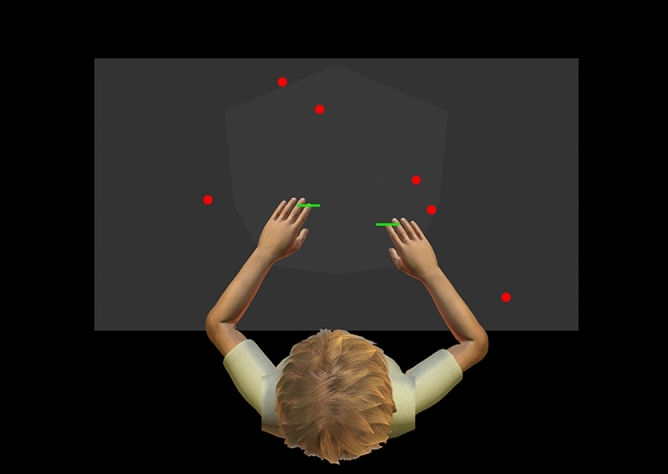
Schematic of the object hit task virtual workspace. Green paddles were displayed on participants fingertips. Participants then attempted to hit as many red balls away from them as possible, as the balls were dropped from the top of the screen.

On the virtual display screen, 5 cm green paddles were displayed at participants’ fingertips (Fig. 2). Balls fell from the top of the screen, from 1 of 10 spatial bins located equally across the display, and participants were instructed to “hit as many balls as possible with either hand”. A total of 300 balls were dropped over 2 minutes and 18 seconds. Balls that were successfully hit would follow a ballistic trajectory, leaving the workspace area; balls that were missed would continue to fall, leaving the workspace area at the bottom of the screen. As time progressed, balls were dropped with increasing speed and frequency. Task performance was measured at a sampling frequency of 200 Hz using five selected parameters, which are outlined below. This object hit task has been described in detail previously^29,30^.

#### Measured parameters

1. *Hits with dominant and non-dominant hands (number)*—quantifies the number of balls successfully hit with each limb; a successful hit occurs when a ball is hit and leaves the display area at the top or sides of the workspace.
2. *Movement area ( in meters*^*2*^*)*—measure of the spatial area used by the dominant and nondominant limbs during the task.
3. *Movement speed (in meters/second)*—measure of the average hand speed of the dominant and nondominant limbs during the task. Hand speed was filtered using a sixth-order double-pass Butterworth filter with a cut-off frequency of 10 Hz.
4. *Hand bias of hits*—identifies the relative limb use (dominant versus non-dominant hand) calculated as ((dominant hits − non-dominant hits)/(dominant hits + non-dominant hits)); a score of 0 indicates that the participant hit balls equally with both the dominant and non-dominant limbs; a negative score indicates that the participant hit more balls with their non-dominant limb; a positive score indicates that the participant hit more balls with their dominant limb.
5. *Hand transition (in meters)*—identifies the spatial location within the workspace where a participant’s preference switches from one limb to the other; a score of 0 indicates that the participant switched from one arm to the other at the center of the workspace, whereas a positive score indicates that the participant crossed over into the non-dominant limb workspace with their dominant limb.

### Statistical analysis

Motor performance was first investigated at the individual level using MATLAB 2015a (The Mathworks, Inc., Nantick, MA). For each parameter outlined above, we determined age predicted typical performance ranges by fitting 95% prediction bands to the scores of all control children (N = 155). We then visualized performance of each child with DCD with respect to these curves; specifically, DCD participants’ performance scores were plotted onto each age-predicted curve. DCD children who fell outside of these 95% age-predicted performance bands were considered impaired on that parameter.

**Figure 3:**
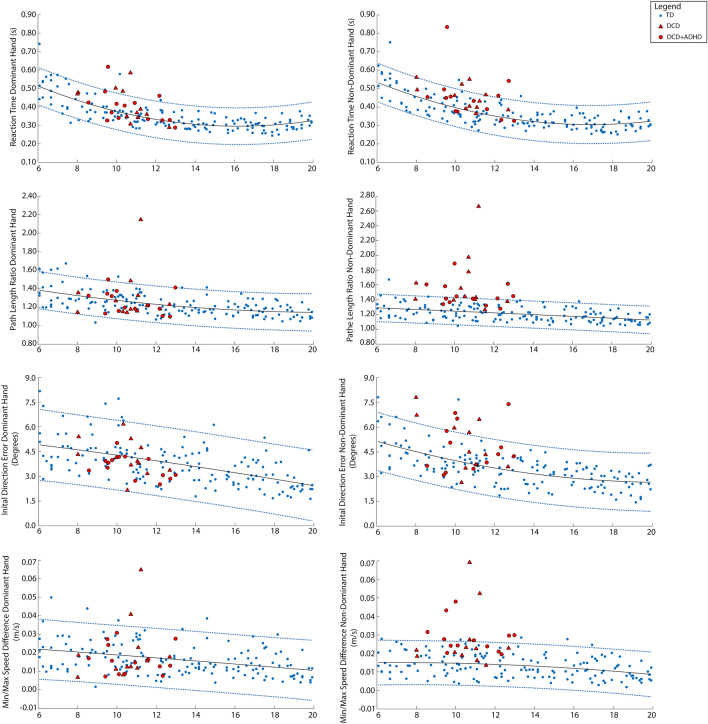
Individual performance of children with DCD on the visually guided reaching task. Age curves and 95% prediction bands based on performance of all TD participants (n = 155) are shown in blue. Performance scores of each DCD participant (n = 26) are superimposed and shown in red (triangles represent DCD participants without ADHD and circles represent DCD participants with co-occurring ADHD).

**Figure 4:**
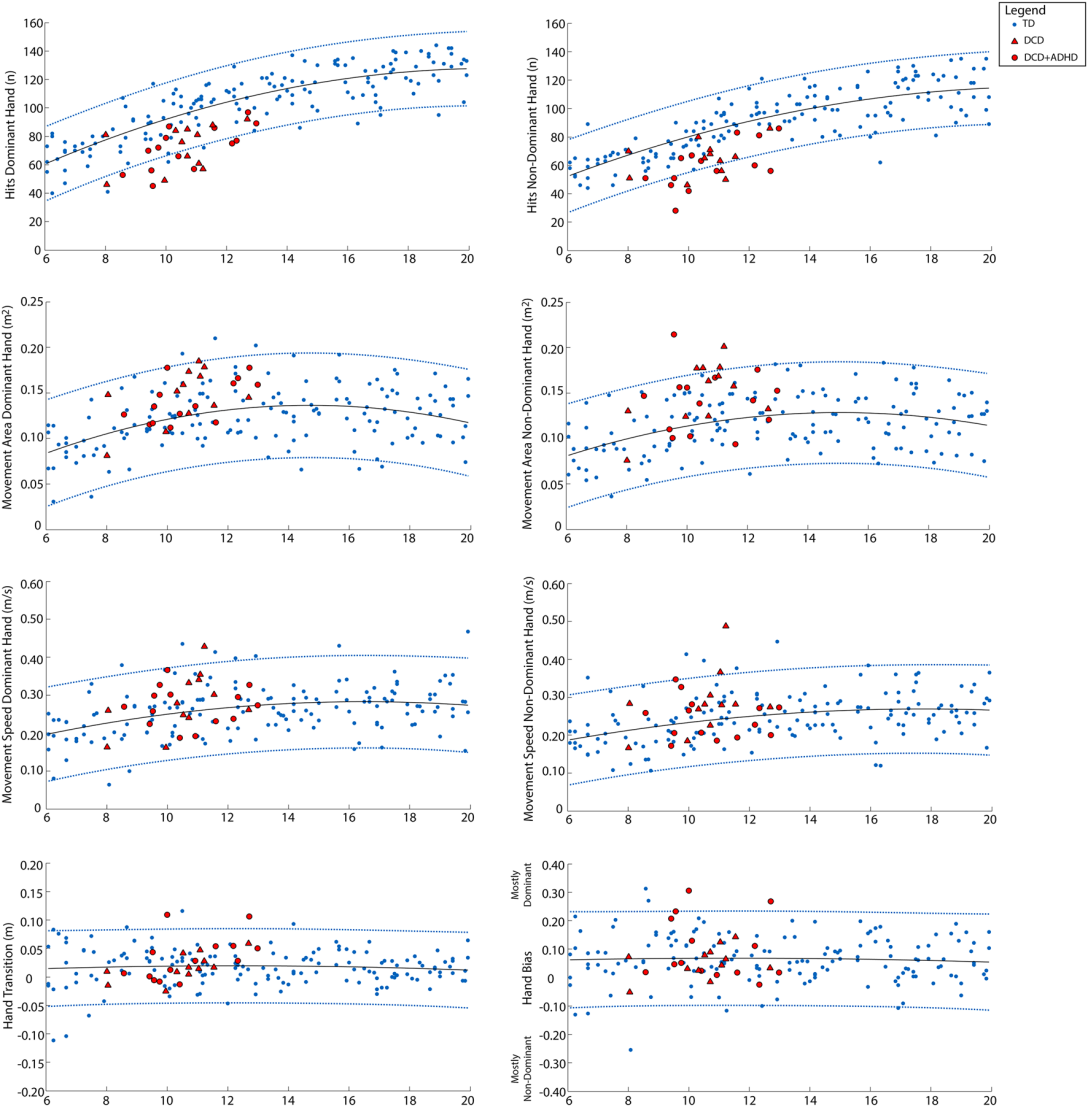
Individual performance of children with DCD on the bimanual object hitting task. Age curves and 95% prediction bands based on performance of all TD participants (n = 155) are shown in blue. Performance scores of each DCD participant (n = 26) are superimposed and shown in red (triangles represent DCD participants without ADHD and circles represent DCD participants with co-occurring ADHD).

Next, group level analyses were performed in SPSS (IBM SPSS Software, V25)^42^. A subsample of controls (n = 57) within the same age range as the DCD group (8–12 years) were used in the group analyses, given the effect of age on the developmental trajectories of each parameter (see results from individual level analyses). An independent samples t-test was used to confirm the absence of mean age differences between the DCD group and the subsample of controls (see Supplementary Table 1 for a detailed breakdown of age frequencies, ranges, means and medians by group), and a chi-square test was used to investigate the sex distribution between the two groups. Given that our KINARM metrics were not normally distributed, Kruskal–Wallis tests, using age-adjusted residuals, compared the performance of children with DCD to that of the subsample of controls on each parameter. Age adjusted residuals were used given the significant effect of age on each KINARM metric (see Figs. 3 and 4).

Finally, as a secondary analysis, using SPSS (IBM SPSS Software, V25.0), Spearman Partial correlations were run to examine the associations between each kinematic parameter and MABC-II Total Test and subscale standard scores in children with DCD, controlling for age.

## Results

### Participants

Group demographics and clinical characteristics are shown in Table 1. There were no significant differences in age (t = 0.244, *p* = 0.807) or sex (*p* = 0.116) between DCD participants and the subsample of controls (n = 57) included in the group analysis. None of the DCD participants were left-handed, as per inclusion criteria. However, 13 of the 155 controls were left-handed and of the controls included in the group level analysis (n = 57), 6 were left-handed.

**Table 1:** Participant demographics and clinical characteristics.

	DCD	TD (all participants)	TD (subsample)
N	26	155	57
Sex (Male:Female)	20:6	81:74	32:25
Age (mean years ± SD)	10.6 ± 1.3	12.5 ± 4.0	10.5 ± 1.3
**MABC-II (mean standard score ± SD)**			
Total test	3.16 ± 1.70	–	–
Manual dexterity	4.04 ± 1.75	–	–
Aiming and catching	5.04 ± 2.37	–	–
Balance	4.58 ± 2.25	–	–
WASI-II Full-Scale IQ (mean ± SD)	102 ± 12	–	–
**Co-occurring diagnoses (N (%))**			
Attention deficits	14 (54)	–	–
Learning disorders	10 (38)	–	–
Anxiety disorder	4 (15)	–	–

### Clinical characteristics of the DCD group

As per the inclusion criteria, all DCD participants displayed motor deficits on the MABC-II, with Total Test scores falling below the 16th percentile (mean Total Test Standard score 3.16; standard deviation: ± 1.72). Additionally, all DCD participants met criteria for typical cognitive performance with FSIQ scores $\ge$ 80 (mean FSIQ: 102; standard deviation: ± 12, range 80–135).

### Individual performance of children with DCD

Age curves and 95% prediction bands based on performance of all control participants (n = 155) are shown in Fig. 3, for the visually guided reaching task, and Fig. 4, for the object hit task, with DCD participants (n = 26) superimposed. Participants with DCD falling outside of the 95% prediction bands developed from the control data were classified as being impaired on a given parameter.

For the visually guided reaching task, impairments were more frequent in the non-dominant limb compared to dominant limb. Five children with DCD (19%) were classified as impaired on the reaction time parameter with their dominant limb and 7 (27%) with their non-dominant limb. Notably, for the path length ratio parameter, 4 children with DCD (15%) were impaired on the dominant limb and 14 (54%) on the non-dominant limb. No children with DCD were impaired on the initial direction error parameter for their dominant limb; however, 8 (31%) were impaired with their non-dominant limb. Finally, for min–max speed difference, 2 (8%) children with DCD were impaired with their dominant limb and 10 (38%) with their non-dominant limb.

For the object hit task, 12 (46%) children with DCD were classified as impaired on the number of balls hit with their dominant limb and 10 (38%) with their non-dominant limb. For the movement area parameter, only 1 DCD participant fell below the 95% prediction band with their dominant limb; however, 5 (19%) DCD participants fell above the 95% band with their non-dominant limb, indicating that DCD participants were moving over a larger workspace area than controls with their non-dominant limb but still hitting fewer balls. Similarly, only 1 DCD participant was impaired on the movement speed parameter for the dominant limb; however, 3 (12%) were impaired with the non-dominant limb. Finally, few children with DCD were impaired on the hand transition parameter (n = 2; 8%) or the hand bias of hits parameter (n = 2; 8%). Specifically, the two children that did show impairments on the hand transition and hand bias of hits parameters, hit more balls with their dominant limb and utilized the workspace area on the dominant limb side more than the workspace area of the non-dominant side.

Supplementary Table 2 demonstrates that DCD participants falling outside the 95% prediction bands primarily showed impairments on 1–2 kinematic parameters for each robotic task, with very few participants showing impairments on more than three task parameters. No DCD participants showed impairments across all parameters. Finally, 3 DCD participants fell within the 95% prediction bands on all task parameters.

### Group differences in performance

**Figure 5:**
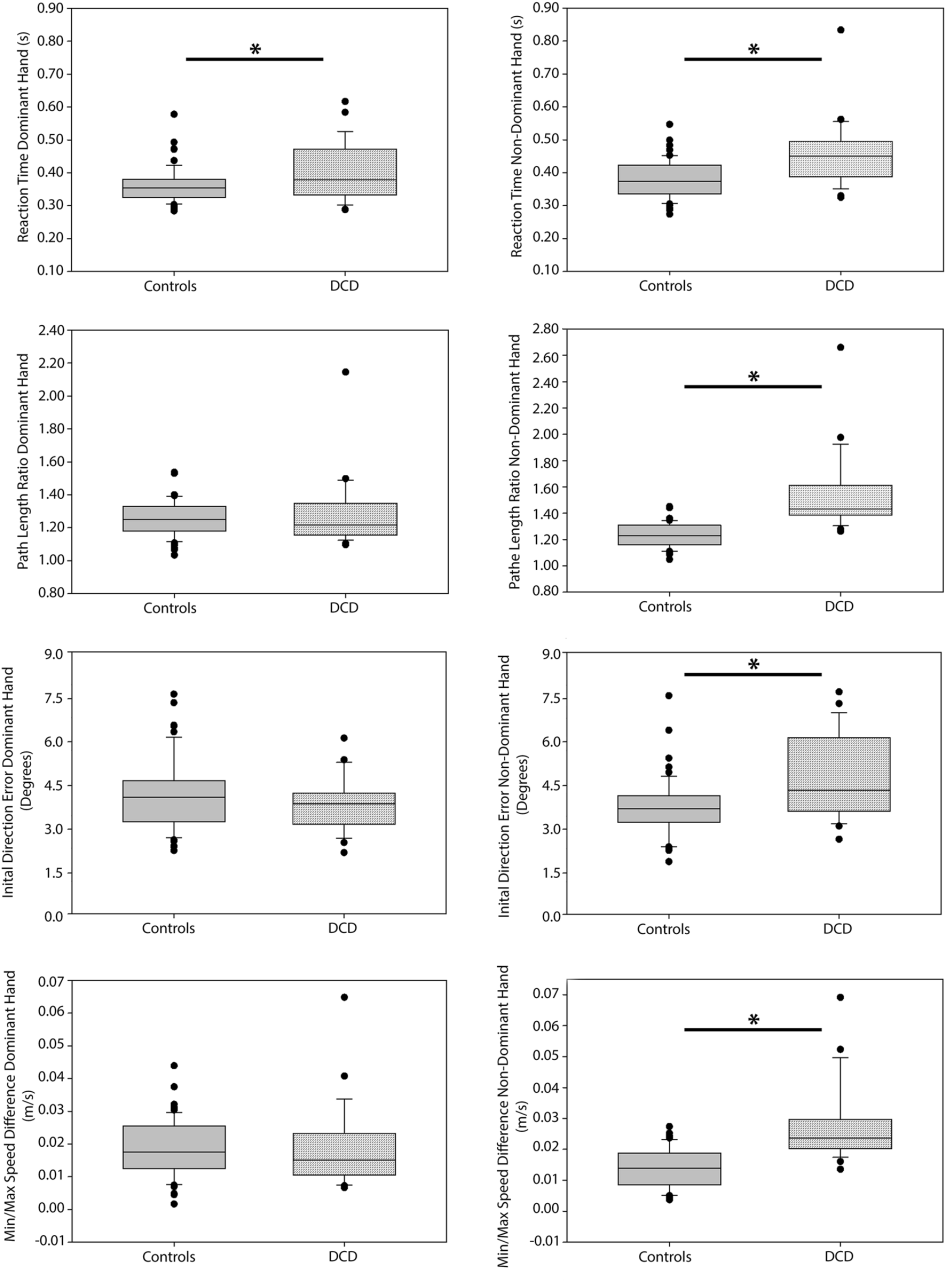
Group differences in performance on the visually guided reaching task. Box plots are shown for both the dominant and non-dominant limbs. *p < 0.05.

When investigating performance differences on the visually guided reaching task between DCD participants and the subsample of controls (n = 57), significant group differences were seen on all parameters for the non-dominant limb (reaction time: H = 17.097, df = 1, *p* ≤ 0.001, partial eta^2^ = 0.217; path-length ratio: H = 41.883, df = 1, *p* ≤ 0.001, partial eta^2^ = 0.969; initial direction error: H = 11.149, df = 1, *p* = 0.001, partial eta^2^ = 0.115; min–max speed difference: H = 31.534, df = 1, *p* ≤ 0.001, partial eta^2^ = 0.565) (see Fig. 5). Specifically, DCD participants demonstrated slower reaction times, as well as poorer accuracy (i.e., greater deviation from an ideal path trajectory) and greater variability in movement speed compared to controls. For the dominant limb significant group differences were only observed for reaction time (H = 7.079, df = 1, *p* = 0.023, partial eta^2^ = 0.054); DCD participants showed slower responses to peripheral targets than controls. No group differences were noted for the initial direction error, min–max speed or path-length ratio parameters for the dominant limb (*p* > 0.1).

**Figure 6:**
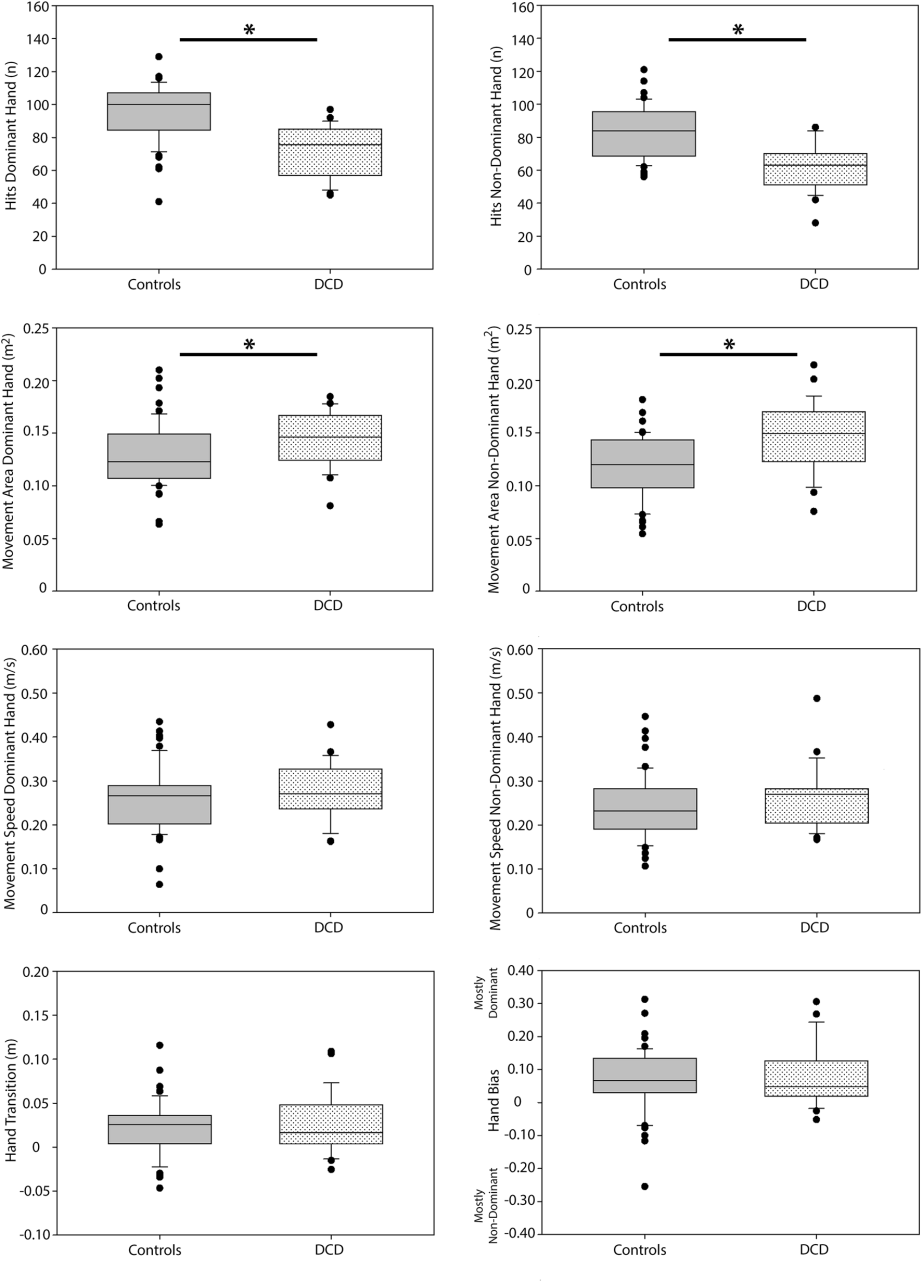
Group differences in performance on the bimanual object hitting task. Box plots are shown for both the dominant and non-dominant arms. *p < 0.05.

Significant group differences between DCD participants and the subsample of controls on the object hitting task were observed for total hits (dominant: H = 38.381, df = 1, *p* ≤ 0.001, partial eta2 = 0.793; non-dominant: H = 34.241, df = 1, *p* ≤ 0.001, partial eta^2^ = 0.641) and movement area (dominant: H = 11.341, df = 1, *p* = 0.023, partial eta^2^ = 0.116; non-dominant: H = 5.786, df = 1, *p* ≤ 0.001, partial eta^2^ = 0.036) (see Fig. 6). Specifically, DCD participants hit fewer balls with their dominant and non-dominant limbs while also using more of the workspace area. No group differences were noted for the movement speed parameter for either the dominant or non-dominant limb (*p* > 0.1), or for the hand transition and hand bias of hits parameters (*p* > 0.1).

### Relationships between clinical and robotic measures in children with DCD

Test statistics for Spearman partial correlations are shown in Supplementary Table 3. For the visually guided reaching task, MABC-II Aiming and Catching subscale scores were negatively correlated with non-dominant limb reaction time; specifically, higher scores on this MABC-II subtest, which indicated better performance, were significantly correlated with faster (i.e., lower) reactions times on the visually guided reaching task. MABC-II Balance subscale scores were negatively associated with dominant limb min/max speed difference and non-dominant limb initial direction angle; such that lower scores on the MABC-II, indicating poorer performance, were associated with larger variability in speed (greater min/max speed differences) and larger deviations from an ideal path trajectory (higher initial direction angle).

For the bimanual object hitting task, MABC-II Total Test standard scores were positively correlated with the total number of balls hit with the non-dominant limb, such that better performance on the MABC-II (a higher score) was associated with better performance on the object hitting task (a greater number of balls hits). No other associations were observed between kinematic parameters and MABC-II scores. All findings would not remain significant if corrected for multiple comparisons; however, this study was not powered to assess this.

## Discussion

Reaching paradigms have been widely used in the literature to investigate motor control in children with DCD^4^; however, studies have placed an emphasis on the assessment of the dominant limb in affected individuals and have typically investigated group differences in performance. The current study employed a unimanual reaching task and a bimanual object hitting task to investigate dominant limb, non-dominant limb and bimanual motor performance in a DCD population. Our findings are consistent with previous evidence of kinematic deficits, including slower reaction time, reduced accuracy and more variable movement speed when reaching, in children with DCD; however, impairments were frequently more pronounced in the non-dominant limb. On a more difficult bimanual task, bilateral impairments in spatiotemporal metrics were commonly observed in children with DCD.

Here, we investigated task-based motor performance using group and individual approaches. Findings from the group analyses revealed that on the visually guided reaching task, significant differences were noted on all parameters for the non-dominant limb including decreased reaction time, greater trajectory path lengths, greater deviation from an ideal trajectory and more variability in movement speed in the DCD group. Interestingly, for the dominant limb, group differences were only observed for reaction time. These findings indicate that although impairments were observed bilaterally, deficits were more common in the non-dominant limb in children with DCD.

Currently therapy programs for DCD often use task orientated approaches to target functional deficits in the dominant limb (i.e., hand-writing, throwing or catching a ball with the dominant hand)^43^. Given that children with DCD showed significant bilateral impairments and that a greater number of children demonstrated impairments with their non-dominant limb, therapies that place equal emphasis on both dominant and non-dominant limb performance may be essential for improved outcomes; particularly on tasks that demand the coordination of both limbs to attain successful performance. Finally, these results highlight the importance for future research to include assessments of both limbs, as in the current study some children with DCD showed impairments in their non-dominant limb in the absence of any impairments in the dominant limb when reaching. It is possible that the lack of impairment in dominant limb performance could be associated with its regular daily use in activities of daily living.

The bimanual object hitting task used here, provided an opportunity to examine limb interactions as well as differences in limb use and/or limb performance during a task of heightened difficulty that demanded greater cognitive and visuospatial attention. Overall, the DCD group hit significantly fewer balls than controls with their dominant and non-dominant limbs, despite using more of the workspace area. These findings provide further support that children with DCD display bilateral impairment. There was, however, no significant difference in hand bias between the DCD group and controls, indicating that both children with DCD and control children hit more balls with their dominant limb. Additionally, the DCD group transitioned from their dominant to non-dominant limb at a spatial location similar to controls, suggesting no asymmetry in limb preference or use. It is important to note that the current object hitting task examined bilateral coordination with independent goals of each limb. Future studies that examine bilateral coordination with a shared goal for the dominant and non-dominant limbs could help to further elucidate bimanual coordination impairments in children with DCD.

As mentioned above, previous reaching studies in children with DCD have focused primarily on group level differences in performance. However, given the significant variability in motor performance that has been reported among children with DCD in the literature^16,44,45^, the results from group level analyses may not apply to individual children. Therefore, one key strength of the current study was the inclusion of a large control population, which allowed for us to establish typical performance ranges across childhood and adolescence on each metric and examine the performance of children with DCD at the individual level. Visualization of performance curves for each metric revealed that varying percentages of children with DCD (8–54%) fell outside the typical performance range of the controls. Correspondingly, this indicated that several children with DCD performed within the age predicted ranges on various measures, providing strong support to the heterogeneous nature of the disorder^5^. These findings highlight the importance for future research to assess performance at both an individual and group level, and to include more comprehensive assessments of children with DCD. Specifically, it may be important for future research to include tasks of varying difficulty that measure both dominant and non-dominant limb motor performance when assessing motor control in children with DCD.

The MABC-II is a standardized assessment tool commonly used by healthcare providers and researchers to assess motor function in children with DCD^2^. Given this, we investigated the associations between each kinematic parameter and children’s MABC-II Total Test scores, as well as the subscale standard scores in our DCD sample; this analysis was done to determine if motor performance on each robotic task was associated with scores on this clinical assessment tool. However, limited associations were observed. Specifically, for the visually guided reaching task, better performance on the MABC-II Aiming and Catching subscale was associated with faster non-dominant limb reaction times. Poorer performance on the MABC-II Balance subscale was associated with larger variability in speed of the dominant limb as well as larger deviations from an ideal path trajectory of the non-dominant limb. For the bimanual object hit task, better overall performance on the MABC-II was associated with a greater number of balls hit with the non-dominant hand. No other associations were observed.

The finding of limited associations between the MABC-II and the robotic assessment measures is not unexpected as these assessments investigate different motor constructs. The MABC-II assesses children’s abilities to perform specific functional motor tasks such as throwing a ball at a target, tracing a trail and constructing a triangle. It does not specifically quantify how the child successfully accomplishes these tasks. In contrast, the robotic measures utilized in the current study allowed for the quantification of children’s motor performance, informing underlying impairment as opposed to function. The robotic assessment tasks demonstrated that children with DCD displayed slower movement initiation, poorer accuracy and greater variability in movement speed compared to controls. These findings provide insight into underlying motor control deficits among children with DCD that could affect their performance on functional motor tasks. A better understanding of these deficits could be used to inform task-oriented interventions for DCD recommended in recently published guidelines^2^. For instance, interventions that target a specific attribute of motor control (i.e., motor speed) could certainly be integrated into a therapy program focused on restoring function in children with DCD.

It is important to note that many children in the DCD group had additional diagnoses, including attention, learning, and anxiety disorders, which could have had an effect on their performance. However, our findings revealed that both children with DCD and children with DCD and co-occurring ADHD fell outside the 95% prediction bands of the controls, suggesting that the additional diagnosis of ADHD did not solely explain the impairments seen. Additionally, although our control group did not have any documented diagnoses, many of these children could have undiagnosed attention, learning, or anxiety disorders. Given that DCD frequently co-occurs with other neurodevelopmental disorders (e.g., 50% with ADHD, 35–40% with reading disabilities^46^), future studies that are adequately powered and include detailed screening of both control and DCD participants for neurodevelopmental disorders are needed. Such studies will help to better understand how motor control in children with DCD is affected by co-occurring diagnoses.

## Conclusions

The results of the present study provide new information regarding motor impairment in children with DCD, supporting the presence of underlying deficits in motor control. Findings of dominant and more commonly, non-dominant limb impairment emphasize the importance of examining in detail motor performance of both limbs in children with DCD. This is of particular significance given that much of the current research, as well as interventions for children with DCD, focus primarily on performance or function of the dominant limb. Finally, the current study demonstrates the potential of robotics to assist in understanding and identifying motor impairment in children with DCD.

## Supplementary Information

Supplementary Tables.

## Data Availability

Data available upon reasonable request.
