# Supplementary material for: Unimanual and bimanual motor performance in children with developmental coordination disorder (DCD) provide evidence for underlying motor control deficits
Source: Sci Rep. 2021 Mar 16;11:5982. doi: 10.1038/s41598-021-85391-3 (PMC7971033; doi:10.1038/s41598-021-85391-3)
Supplement: Supplementary file 1 — Supplementary Tables. [file 41598_2021_85391_MOESM1_ESM.docx]

**Supplementary Information**

**Unimanual and bimanual motor performance in children with developmental coordination disorder (DCD): Evidence of underlying motor control deficits.**

Melody N. Grohs^1,2^, Rachel L. Hawe^3,4^, Sean P. Dukelow^3,4^, *Deborah M. Dewey^2,4,5,6^

1 Department of Neurosciences, University of Calgary

2 Owerko Centre at the Alberta Children’s Hospital Research Institute (ACHRI)

3 Department of Clinical Neurosciences, University of Calgary

4 Hotchkiss Brain Institute (HBI), University of Calgary

5 Department of Pediatrics, University of Calgary

6 Department of Community Health Sciences, University of Calgary

***Corresponding Author:**

Dr. Deborah Dewey

Ph: 1(403)441-8468

Em: [dmdewey@ucalgary.ca](mailto:dmdewey@ucalgary.ca)

#397, Child Development Center, 2500 University Dr. NW, Calgary, AB T2N 1N4

**Supplementary Table S1: Age frequencies, ranges, means and medians shown by group.**

|  | **Frequency** | |
| --- | --- | --- |
| **Age** | **DCD (n=26)** | **Controls (n=57)** |
| 8.0 - 8.99 | 3 | 10 |
| 9.0 - 9.99 | 5 | 10 |
| 10.0 - 10.99 | 8 | 13 |
| 11.0 - 11.99 | 5 | 12 |
| 12.0 - 12.99 | 5 | 12 |
| Minimum | 8.02 | 8.08 |
| Maximum | 12.98 | 12.92 |
| Mean | 10.61 | 10.54 |
| Median | 10.61 | 10.50 |

**Supplementary Table S2: Number of DCD and control participants falling outside the 95% prediction bands on 1 or more parameters.**

|  | | **Number of DCD Participants Falling Outside 95% Prediction Bands** | | | | |
| --- | --- | --- | --- | --- | --- | --- |
|  | | **1 Parameter** | **2 Parameters** | **3 Parameters** | **4 Parameters** | **5**  **Parameters** |
| VGR Dominant | | 5 | 3 | -- | -- | -- |
| VGR Non-Dominant | | 7 | 9 | 2 | 2 | -- |
| OH | | 8 | 5 | 3 | 1 | 1 |
|  | **Number of Control Participants Falling Outside 95% Prediction Bands** | | | | | |
|  | | **1 Parameter** | **2 Parameters** | **3 Parameters** | **4 Parameters** | **5**  **Parameters** |
| VGR Dominant | | 4 | 3 | 1 | -- | -- |
| VGR Non-Dominant | | 3 | 1 | -- | -- | -- |
| OH | | 10 | 3 | 4 | -- | -- |

***Note:** Control data is shown for the subsample of controls (n=57) falling within the same age range as DCD participants (8-12 years). VGR: visually guided reaching; OH: object hitting.

**Supplementary Table S3: Test Statistics of Spearman Partial Correlations Examining the Associations of Clinical and Robotic Measures in Children with DCD.** Results from correlations between the MABC-II Standard scores (Total Test, Manual Dexterity (MD), Aiming & Catching (AC), and Balance (B)) and each kinematic parameter on the visually guided reaching task (dominant and non-dominant limbs) and bimanual object hitting task, controlling for age. **p*<0.05.

| **MABC-II Total Test**  (r, *p*) | | **MABC-II MD**  (r, *p*) | **MABC-II AC**  (r, *p*) | **MABC-II B**  (r, *p*) |
| --- | --- | --- | --- | --- |
| **Reaching Dominant Limb** |  |  |  |  |
| Reaction Time | -0.113, 0.589 | 0.020, 0.923 | -0.243, 0.243 | -0.080, 0.702 |
| Initial Direction Error | -0.111, 0.596 | -0.002, 0.992 | 0.066, 0.753 | -0.212, 0.310 |
| Min-Max Speed Difference | -0.267, 0.197 | -0.126, 0.547 | -0.141, 0.502 | **-0.423, 0.035*** |
| Path Length Ratio | -0.255, 0.219 | -0.185, 0.376 | -0.022, 0.915 | -0.372, 0.067 |
| **Reaching Non-Dominant Limb** |  |  |  |  |
| Reaction Time | -0.090, 0.675 | 0.243, 0.253 | **-0.470, 0.020*** | -0.176, 0.411 |
| Initial Direction Error | -0.165, 0.442 | 0.127, 0.554 | 0.065, 0.762 | **-0.415, 0.044*** |
| Min-Max Speed Difference | -0.167, 0.436 | -0.156, 0.467 | 0.028, 0.896 | -0.207, 0.331 |
| Path Length Ratio | -0.218, 0.306 | -0.009, 0.965 | -0.108, 0.615 | -0.314, 0.135 |
|  |  |  |  |  |
| **Bimanual Object Hitting** |  |  |  |  |
| Hand Bias | -0.001, 0.996 | 0.245, 0.238 | -0.117, 0.579 | -0.141, 0.502 |
| Hand Transition | 0.078, 0.711 | 0.142, 0.499 | -0.195, 0.351 | 0.069, 0.743 |
| Hits Dominant | 0.802, 0.408 | 0.189, 0.365 | 0.211, 0.311 | 0.366, 0.072 |
| Hits Non-Dominant | **0.428, 0.033*** | 0.380, 0.061 | 0.320, 0.119 | 0.244, 0.240 |
| Movement Speed Dominant | -0.111, 0.597 | 0.166, 0.427 | -0.073, 0.729 | -0.264, 0.202 |
| Movement Speed Non-Dominant | -0.208, 0.319 | 0.185, 0.375 | -0.175, 0.403 | -0.369, 0.070 |
| Movement Area Dominant | -0.186, 0.374 | 0.010, 0.961 | -0.237, 0.255 | -0.165, 0.430 |
| Movement Area Non-Dominant | -0.314, 0.126 | 0.011, 0.958 | -0.307, 0.135 | -0.353, 0.084 |
|  |  |  |  |  |
